# Severe Small-Bowel Obstruction in a High-Risk Patient on Long-Term Tirzepatide Therapy: A Case Report

**DOI:** 10.7759/cureus.98935

**Published:** 2025-12-10

**Authors:** Shamsun Nahar, Nelly Maybee, Nowrin Tamanna, Anahita Sadat, Farjana Khanam, Rokeya Begum, Sume Akther, Mishma Salsabil Khan, Shamsun Nahar Sonia, Nahid Hasan

**Affiliations:** 1 Internal Medicine, Augusta Health, Fishersville, USA; 2 Internal Medicine, California Institute of Behavioral Neurosciences and Psychology, Fairfield, USA; 3 Diabetes and Endocrinology, Augusta Health, Fishersville, USA; 4 Internal Medicine, Chittagong Medical College, Chattogram, BGD; 5 Epidemiology, University of South Carolina, Columbia, USA; 6 Internal Medicine, Dhaka Medical College Hospital, Dhaka, BGD; 7 Medicine, Central Medical College, Comilla, BGD; 8 Internal Medicine, Jalalabad Ragib-Rabeya Medical College and Hospital, Sylhet, BGD; 9 Internal Medicine, Institute of Applied Health Sciences, Chattogram, BGD; 10 Internal Medicine, Dream USMLE, Chesterfield, USA; 11 Medicine, Dhaka Medical College Hospital, Dhaka, BGD; 12 Internal Medicine, Institute of Applied Health Sciences (IAHS), Dallas, USA

**Keywords:** glp-1/gip therapy, glycemic control, small bowel obstruction, tirzepatide, type 2 diabetes mellitus

## Abstract

The introduction of tirzepatide marks a significant advancement in the management of type 2 diabetes mellitus (T2DM) and obesity. This dual synthetic polypeptide, glucose-dependent insulinotropic polypeptide (GIP), and glucagon-like peptide-1 receptor agonist (GLP-1 RA) acts through incretin pathways and stimulates insulin sensitivity while slowing gastric emptying. While these benefits are well established, the drug’s effects on gastrointestinal motility may predispose susceptible patients to several gastrointestinal complications.

We present a rare case of severe small bowel obstruction in a 61-year-old woman with diabetes and morbid obesity who had been on long-term tirzepatide (Mounjaro) therapy. She presented to our hospital with acute, severe, diffuse abdominal pain, accompanied by dry heaving. She denied any hematemesis, melena, or hematochezia. She was receiving tirzepatide 2.5 mg weekly, then titrated to 5 mg after one month, and this dose was maintained for over a year. Since initiation, her hemoglobin A1c decreased from 8.6% to 5.6%, insulin therapy was discontinued, and she achieved significant weight loss, with improved liver function and laboratory parameters. However, she developed progressive constipation, with only one to two bowel movements per week, representing a marked change from her baseline. CT scans of the patient’s abdomen and pelvis revealed multiple dilated, fluid-filled small bowel loops, with a transition point in the midline lower pelvis. There was also moderate gastric distention and moderate free fluid in the pelvis. No evidence of perforation or mass was seen. The patient was initially managed with nasogastric decompression, bowel rest, intravenous fluids, and analgesia. As her symptoms persisted and repeated examinations raised concern for peritonitis, she underwent laparoscopic adhesiolysis, which was converted to exploratory laparotomy due to dense adhesions. Intraoperatively, a closed-loop obstruction was identified, caused by adhesive disease and an internal hernia, with 25 cm of necrotic small bowel requiring resection. Pathologic evaluation of the resected specimen revealed edema, congestion, and ischemic changes. Although adhesive disease was the direct cause of the obstruction, the temporal relationship between tirzepatide (Mounjaro) initiation, progressive constipation, and eventual obstruction suggests a contributory role.

Our case suggests tirzepatide-induced motility changes may worsen baseline constipation and precipitate obstructive complications in predisposed patients. Clinicians should monitor bowel function closely in high-risk patients and have a low threshold for imaging in the presence of obstructive symptoms.

## Introduction

In recent years, the close relationship between obesity and type 2 diabetes mellitus (T2DM) has become increasingly evident. National data from 2017 to 2020 show that nearly 90% of U.S. adults diagnosed with T2DM were classified as overweight or obese (body mass index (BMI) ≥ 25 kg/m²). Because of this strong association, the American Diabetes Association (ADA) has recommended obesity as the primary screening criterion for identifying individuals at risk for diabetes and prediabetes [1]. This connection has also underscored the importance of addressing excess weight as a cornerstone of T2DM prevention and management. While lifestyle modifications, such as diet and exercise, remain first-line strategies, additional therapeutic options are increasingly necessary, especially for individuals with obesity-related comorbidities or inadequate response to lifestyle alone.

Among these, glucagon-like peptide-1 receptor agonists (GLP-1 RAs) have emerged as a transformative class of medications. Initially developed solely for the management of diabetes, GLP-1 RAs are now widely recognized for their effectiveness in reducing body weight [2]. GLP-1 itself is an incretin hormone secreted by intestinal L cells, with secretion rising several-fold after food intake. GLP-1 receptors are distributed in pancreatic β-cells, the central nervous system, and various peripheral tissues. Through these pathways, GLP-1 regulates insulin secretion, suppresses glucagon release, slows gastric emptying, and influences appetite and satiety. In individuals with T2DM, secretion of GLP-1 is preserved; however, its function is impaired, resulting in reduced insulin response, increased insulin resistance, and poor glycemic control.

In the setting of obesity, GLP-1 plays a critical role in suppressing appetite, lowering food intake, and enhancing satiety, mechanisms that collectively support weight reduction. These physiological effects explain why GLP-1 RAs, including agents such as semaglutide and tirzepatide, have demonstrated dual benefits in both glycemic control and weight management [3,4]. In contrast to GLP-1, glucose-dependent insulinotropic polypeptide (GIP) is secreted by K-cells in the proximal small intestine and also plays a key role in postprandial glucose regulation and body weight. While GIP alone has limited therapeutic use in T2DM due to impaired responsiveness, combining GIP agonism with GLP-1 activity produces synergistic metabolic effects in medications that have dual effects, such as tirzepatide.

More recently, novel incretin-based therapies, such as retatrutide and orforglipron, have shown even greater weight loss outcomes, including in non-diabetic adults [5]. As their use expands beyond diabetes to broader indications in weight management, recognition of their therapeutic potential must also be balanced with awareness of their safety profile. Literature indicates that GLP-1 RAs exert their benefits partly by delaying gastric emptying, enhancing insulin secretion, and suppressing glucagon release. While these mechanisms make them highly effective, they also predispose patients to gastrointestinal adverse effects, such as nausea, vomiting, abdominal discomfort, and constipation. Rare but severe complications, including bowel obstruction, have also been reported, raising important safety considerations [4,6,7].

Although tirzepatide, by being a dual GIP and GLP-1 RA, works on both receptors to affect motility, which is reflected in common side effects like delayed gastric emptying, this case report underscores the need for close monitoring of patients treated with GLP-1 RAs - particularly tirzepatide - and focuses on the GLP-1-mediated effects of this dual GLP-1/GIP agonist, as rare but potentially life-threatening gastrointestinal complications can occur. Although these events are uncommon, their ability to present as surgical emergencies underscores the need for heightened awareness, as delayed recognition can lead to significant morbidity and mortality. Therefore, as the clinical use of incretin-based therapies continues to expand, careful patient selection, ongoing monitoring, and prompt identification of complications will be essential to ensuring safe and effective treatment.

## Case presentation

A 61-year-old woman with a history of T2DM (diagnosed in 2012), cryptogenic cirrhosis (stage IV, with esophageal varices), chronic kidney disease stage III (baseline creatinine 1.3-1.5 mg/dL), hypertension, obstructive sleep apnea, and morbid obesity (BMI 34 kg/m²) presented with acute, severe, diffuse abdominal pain and dry heaving. The pain began abruptly the previous evening, initially attributed to gas, and was unrelieved by simethicone or antacids. She denied any vomiting, hematemesis, melena, or hematochezia. She reported chronic constipation, with bowel movements only once or twice weekly for several months before admission, a change from her baseline of daily to every-other-day bowel movements. 

Her diabetes had been poorly controlled before tirzepatide initiation, with A1c values ranging from 7.2% to 13.7% between 2019 and early 2023. Tirzepatide was started at 2.5 mg weekly in July 2023 and titrated to 5 mg weekly by August 2023, with this dose maintained for over a year. Following initiation, her A1c improved from 8.6% (January 2023) to 6.4% (October 2023), and subsequently to 5.6% (July and November 2024), accompanied by significant weight loss and improved liver function parameters (total bilirubin 0.8, direct bilirubin 0.2, indirect bilirubin 0.3; aspartate aminotransferase 27, alanine aminotransferase 20, alkaline phosphatase 59). She was able to discontinue all prandial and basal insulin after starting tirzepatide. The patient was also following a diabetic diet. Her other home medications included metformin ER, losartan, furosemide, carvedilol, and several neuropsychiatric and pain agents.

Her past medical history is notable for cryptogenic cirrhosis, CKD stage III, hypertension, obstructive sleep apnea, morbid obesity, and a remote episode of Guillain-Barré syndrome (GBS), with residual mild lower extremity weakness. She also has a history of diabetic neuropathy. Her surgical history includes a remote hysterectomy, after which she developed a postoperative small bowel obstruction that was managed conservatively with nasogastric tube decompression, bowel rest, and fluid resuscitation without the need for surgical intervention. Additional surgeries include cesarean section and laparoscopic cholecystectomy. She had no history of tobacco, alcohol, or illicit drug use.

**Figure 1:**
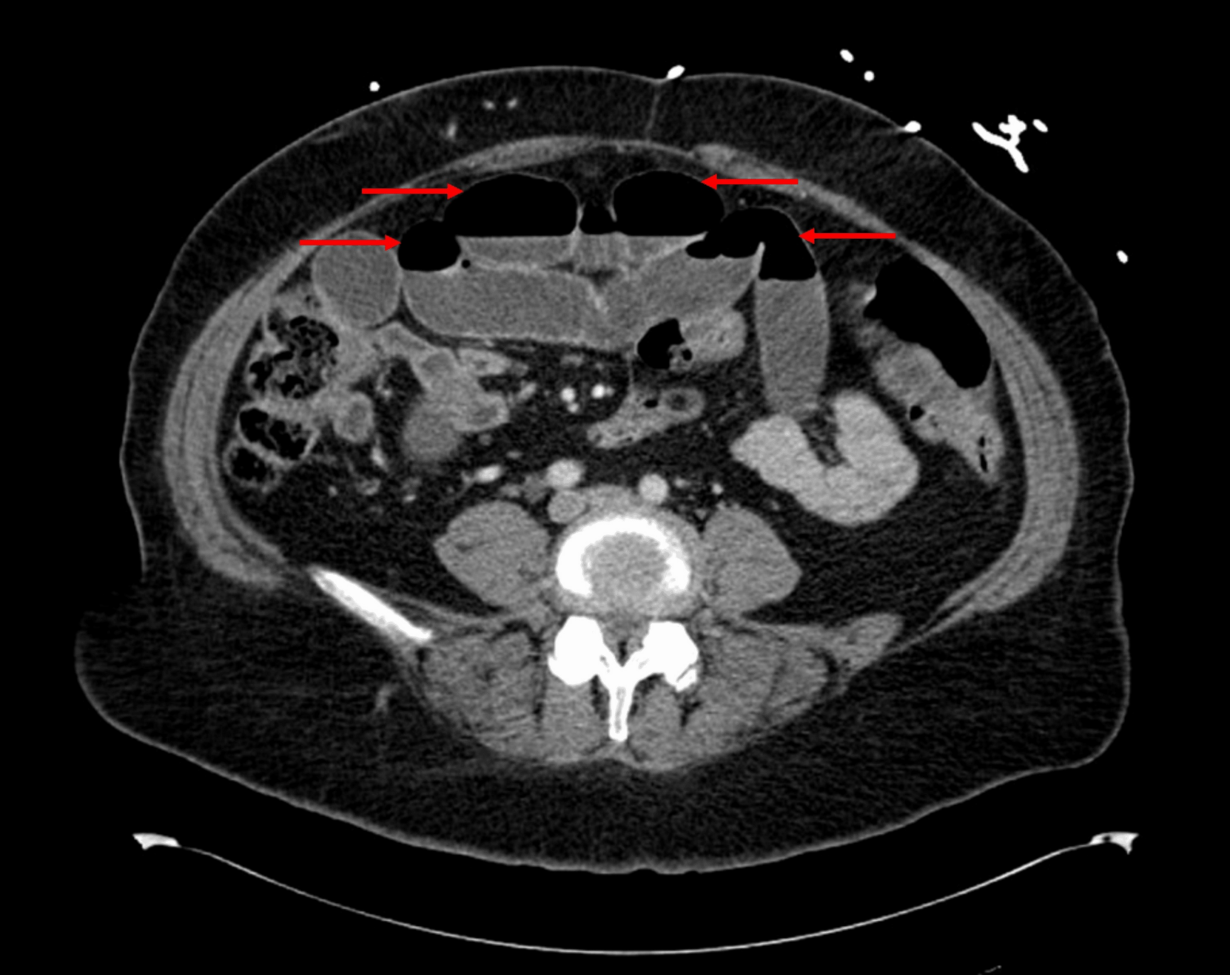
CT scan (axial view) showing dilated loops of bowels (shown by red arrows)

**Figure 2:**
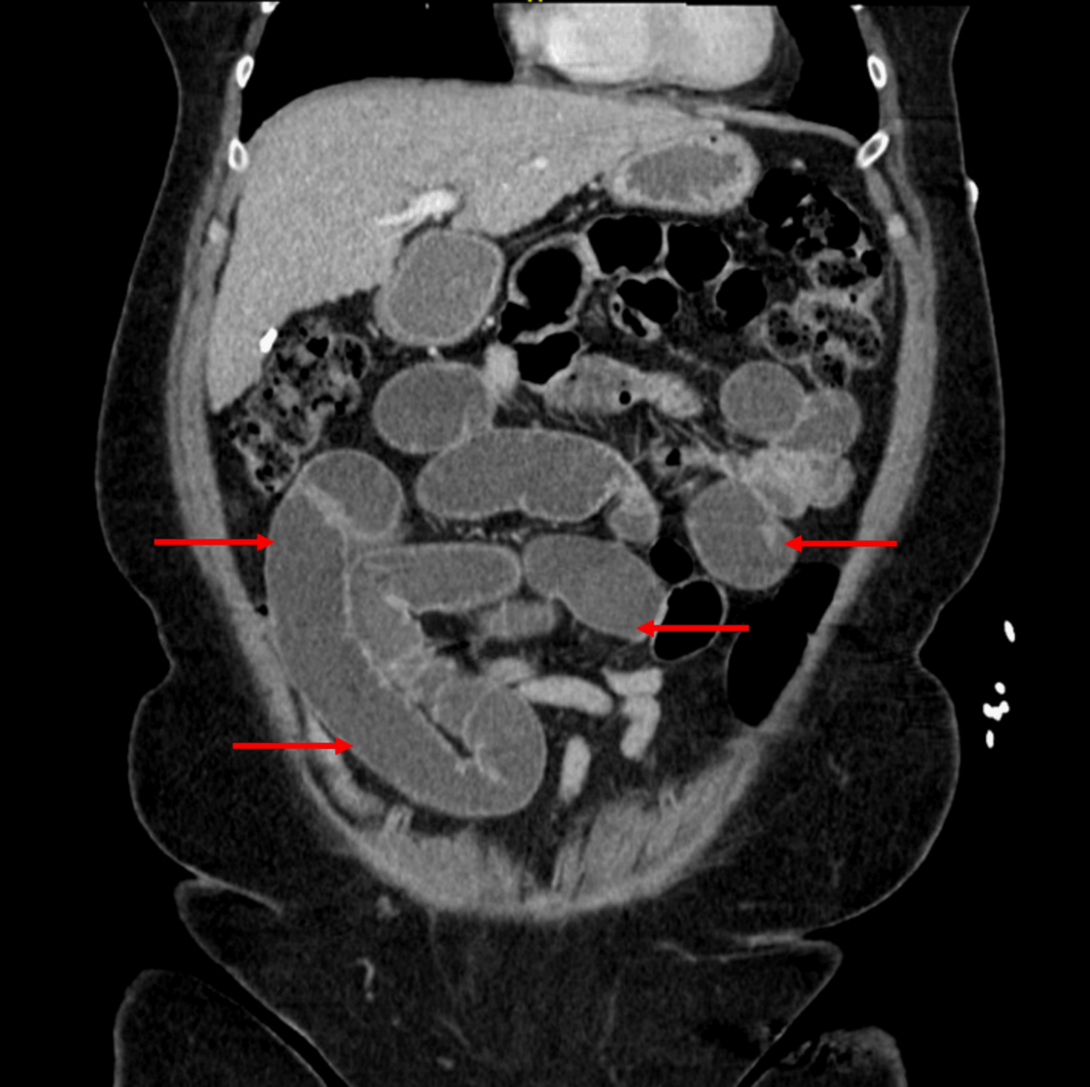
CT scan (coronal view) showing dilated loops of bowel (shown by red arrows)

On presentation (November 2024), she was afebrile, hypertensive (186/85 mmHg), and mildly tachycardic. Abdominal examination revealed obesity, infraumbilical and left lower quadrant tenderness, and hypoactive bowel sounds without peritoneal signs. Laboratory studies showed leukocytosis (WBC 10.3 × 10³/μL), hemoconcentration (hemoglobin 19.4 g/dL, hematocrit 55.5%), low-normal potassium, and CKD stage 3 (creatinine 1.4 mg/dL, eGFR 43 mL/min/1.73 m²). Liver enzymes were within normal limits; however, imaging revealed cirrhotic morphology. CT of the abdomen and pelvis revealed multiple dilated, fluid-filled small bowel loops with a transition point in the midline lower pelvis, moderate gastric distention, and moderate free fluid in the pelvis. No evidence of perforation or mass was seen. Although her hemoglobin and hematocrit appeared markedly elevated on admission, these values were consistent with her chronically borderline-high baseline. The findings were attributed to relative hemoconcentration from dehydration and third-spacing due to small bowel obstruction rather than true polycythemia. She had no symptoms of hyperviscosity, and her levels improved with intravenous fluids; therefore, no emergent hematologic intervention was indicated. A component of chronic secondary erythrocytosis, possibly related to chronic obstructive sleep apnea, may have contributed to her baseline elevations. Table 1 summarizes the laboratory findings on admission alongside reference ranges. Figure 1 (axial view) and Figure 2 (coronal view) show small bowel obstruction on CT, with red arrows indicating the dilated loops of bowel.

**Table 1:** Laboratory results upon admission with reference ranges

Investigation Name	Result	Reference Range
While Blood Cells (WBCs)	10.3 × 10^3^/μL	3.5-10.5 × 10^3^/μL
Hemoglobin	19.4 g/dL	11.6-15 g/dL
Hematocrit	55.50%	36%-44%
Potassium	3.5	3.5-5.2
Creatinine (Cr)	1.4 mg/dL	0.5-1.1 mg/dL
Estimated Glomerular Filtration Rate (eGFR)	43 mL/min/1.73 m^2^	85 mL/min/1.73 m^2^
Total Bilirubin	0.9 mg/dL	0.1-1.2 mg/dL
Aspartate Aminotransferase (AST)	33 U/L	8-43 U/L
Alanine Transaminase (ALT)	42 U/L	-36 U/L
Alkaline Phosphatase (ALP)	79 U/L	30-120 U/L

Initial management included nasogastric decompression, intravenous fluids, bowel rest, and pain control. Despite conservative therapy, the patient’s symptoms persisted, and repeat examination raised concern for evolving peritonitis. She underwent laparoscopic adhesiolysis, which was converted to exploratory laparotomy due to dense adhesions and intraoperative findings. A closed-loop small bowel obstruction was identified, caused by an internal hernia from adhesive disease, with 25 cm of necrotic small bowel requiring resection. The omentum was densely adherent and friable, with significant ascites and cirrhotic liver morphology. A pelvic drain was placed due to ongoing oozing. The pathology of the resected specimen revealed benign small bowel with mucosal and submucosal edema, congestion, and ischemic changes, and the omentum and resection margins were viable.

Postoperatively, the patient required aggressive diuresis with furosemide and spironolactone for her cirrhosis-related ascites, as well as potassium supplementation. She experienced mild hypotension and transient hypokalemia, necessitating close monitoring. Her postoperative course was complicated by prolonged ileus and high drain output, consistent with ascites. She was advanced to a low-residue diet as bowel function returned, and her surgical incision healed without complication. She was discharged on hospital day 18, with close outpatient follow-up, continued diuretic therapy, and avoidance of GLP-1 RAs pending further evaluation of bowel health.

Endocrinology follow-up was arranged at discharge. Given the close temporal relationship, the WHO-UMC causality criteria classified the event as "possible," and the Naranjo scale yielded a score of 4, indicating a possible adverse drug reaction. Therefore, tirzepatide was discontinued. Her diabetes regimen was transitioned to basal insulin (insulin glargine), metformin, and, later, Janumet XR (sitagliptin/metformin) and dapagliflozin, with ongoing continuous glucose monitoring (CGM). The endocrinologist advised close monitoring of glycemic control and weight, and deferred any future GLP-1 RA therapy until bowel health was stable.

Several factors in her medical history predisposed her to the development of small bowel obstruction. Her prior abdominal surgeries, particularly her hysterectomy, complicated by postoperative small bowel obstruction, increased her risk for adhesive disease and subsequent bowel obstruction. The presence of cirrhosis with ascites likely contributed to increased intra-abdominal pressure and the formation of adhesions, further elevating her risk. Chronic kidney disease may have contributed to her electrolyte disturbances and impaired her ability to compensate for acute illness. While her history of GBS and diabetic neuropathy could theoretically contribute to gastrointestinal dysmotility, there was no clear evidence of a primary motility disorder in this case, and the acute presentation was more consistent with a mechanical obstruction rather than a neuropathic or myopathic process. Additionally, her medication regimen - including amitriptyline, gabapentin, duloxetine, citalopram, quetiapine, and tramadol - likely compounded her baseline risk for constipation and impaired GI motility, further predisposing her to obstruction.

The use of tirzepatide (Mounjaro), a GLP-1/GIP RA, may have further contributed to her presentation. GLP-1 RAs are known to delay gastric emptying and can exacerbate constipation, particularly in patients with pre-existing risk factors, such as prior abdominal surgery, adhesive disease, cirrhosis with ascites, and the use of multiple constipating medications. In this patient, the effect of tirzepatide on GI motility likely acted synergistically with her other risk factors, tipping a compensated state of chronic constipation and adhesive disease into a clinically significant, refractory small bowel obstruction. While GLP-1 agonists alone are rarely associated with mechanical obstruction, their impact on motility and transit time can unmask or worsen underlying adhesive disease, especially in complex patients with multiple predisposing factors.

The differential diagnosis for her acute abdominal pain included small bowel obstruction, bowel ischemia, perforated viscus, infectious colitis, and severe constipation or ileus. Small bowel obstruction was strongly suggested by her history of prior abdominal surgery, chronic constipation, and imaging findings of dilated small bowel loops with a transition point. Bowel ischemia was considered, particularly given her hemoconcentration and leukocytosis, and was confirmed intraoperatively by the presence of necrotic bowel. Perforation was excluded by the absence of free air on imaging and the lack of peritoneal signs on initial examination. Infectious colitis was unlikely, given the absence of diarrhea, fever, or leukocytosis out of proportion to her clinical picture. Severe constipation and ileus were considered but were less likely, given the clear mechanical transition point and intraoperative findings. Thus, the diagnosis of adhesive small bowel obstruction with closed-loop ischemia was established.

In summary, this patient’s presentation of severe small bowel obstruction occurred in the context of multiple overlapping risk factors, including prior abdominal surgery with adhesive disease, cirrhosis with ascites, chronic kidney disease, chronic constipation, polypharmacy with constipating agents, and long-term tirzepatide therapy. The interplay of these factors likely contributed to the development of her acute gastrointestinal complication. Notably, the temporal association between tirzepatide initiation, progressive worsening of constipation, and eventual obstruction suggests that GLP-1 RA therapy may have played a significant contributory role in precipitating this event, although it was not the sole etiology. This case underscores the importance of recognizing how GLP-1 RA-induced motility changes can interact with pre-existing risk factors, highlighting the need for careful patient selection, close monitoring, and early intervention in high-risk individuals.

## Discussion

The use of GLP-1 RAs has risen rapidly in the United States because of their dual benefits of glycemic control and weight reduction, with 6% of adults reporting current use in 2024 and prevalence reaching 22% among those advised they were overweight or obese [8]. Nausea, vomiting, and constipation are well-documented adverse events of GLP-1 RAs, with reported incidences ranging from 15%-50%, 5%-20%, and 4%-12%, respectively [9]. Constipation is among the most common gastrointestinal adverse events associated with GLP-1 RAs and is generally regarded as a “yellow-zone” side effect - uncomfortable but typically manageable through dietary modification, hydration, or pharmacologic adjustment. However, when constipation becomes persistent or severe, it may serve as an early indicator of impaired gastrointestinal motility. In susceptible patients, progressive slowing of intestinal transit and fecal stasis can transform a tolerable symptom into a rare but serious “red-zone” complication, such as bowel obstruction. Bowel obstruction manifests with abdominal pain, nausea, vomiting, bloating, and constipation, due to luminal blockage of the intestine. Bowel obstruction represents a critical clinical entity, as it can progress to ischemia, perforation, sepsis, and death if not promptly recognized and managed. This underscores the importance of close monitoring for constipation during GLP-1 RA therapy, not only to preserve patient comfort and adherence, but also to enable early identification of those at risk for life-threatening sequelae [10].

The pathophysiology of GLP-1 RA-associated bowel obstruction is likely multifactorial, with evidence pointing to their strong antimotility properties. GLP-1 RAs inhibit gastric and intestinal motility primarily through actions on myenteric neurons, which modulate gut function via nitrergic and cyclic adenosine monophosphate-dependent pathways. These effects lead to delayed gastric emptying, enhanced pyloric tone, and slowed intestinal transit. The resulting motility changes can create areas of stasis and abnormal peristalsis, which, in turn, may elevate intraluminal pressure and impair luminal clearance. These mechanisms provide a plausible basis for the development of bowel obstruction in susceptible individuals, particularly those with preexisting anatomic or physiological risk factors [11].

Lulu et al. analyzed FAERS data from 2018 to 2022 and identified 21,281 gastrointestinal adverse events among 81,752 reported adverse events associated with GLP-1 RAs. An overall increased risk was observed (ROR 1.46; 95% CI, 1.44-1.49), with the strongest associations seen for semaglutide (ROR 3.00; 95% CI, 2.89-3.11), liraglutide (ROR 2.39; 95% CI, 2.28-2.51), and dulaglutide (ROR 1.39; 95% CI, 1.36-1.42). They further reported significant variation in the severity of gastrointestinal adverse events (p < 0.001), with liraglutide showing the highest severe proportion (23.31%) and dulaglutide the lowest (12.29%), and the majority of the gastrointestinal side effects tended to occur within one month [12].

We recently encountered a similar case at our institution - a 49-year-old woman with type 2 diabetes, obesity, and chronic pain on long-term narcotic therapy, who presented with abdominal distension and vomiting after two years of tirzepatide (Mounjaro) therapy. Her past medical history included prior abdominal surgery for hysterectomy, predisposing her to adhesive disease. CT imaging showed dilated small-bowel loops with a transition point consistent with partial small-bowel obstruction. She was managed conservatively with nasogastric decompression, bowel rest, and intravenous hydration. Her symptoms gradually improved over several days, and she was able to advance to a soft diet without requiring surgery. Given her A1c of 6.4% (in the prediabetic range) and the fact that tirzepatide had been prescribed primarily for weight management by her metabolic clinic - during which she had lost 22 lbs over the recent three months - it was recommended that tirzepatide be discontinued at discharge. She was scheduled for close outpatient follow-up with her primary care physician, metabolic clinic, and endocrinologist for ongoing management of her glucose control and obesity. She remained symptom-free at follow-up. Like the index patient, she had multiple overlapping risk factors - adhesive disease, narcotic use, chronic constipation, and prolonged tirzepatide exposure - suggesting a synergistic effect of impaired motility and structural predisposition. Her relatively milder course underscores that, while not all patients develop severe or ischemic obstruction, the risk spectrum ranges from partial to complete obstruction depending on individual susceptibility.

While postoperative adhesions are the most common cause of small-bowel obstruction in patients with prior abdominal surgeries, emerging clinical reports suggest that GLP-1 RAs may act as additional precipitating factors. These agents are known to delay gastric emptying, enhance pyloric contraction, and alter small-bowel motility, which can contribute to fecal stasis, increased intraluminal pressure, bacterial overgrowth, and, ultimately, mechanical obstruction. Thakkar et al. and Nagib et al. both described semaglutide-associated small-bowel obstruction, whereas Gordon et al. reported a tirzepatide-associated large-bowel obstruction necessitating colectomy [13-15]. Nagib et al. reported a case of a 59-year-old obese woman developing acute small-bowel obstruction shortly after semaglutide administration, whose symptoms of abdominal pain, nausea, and vomiting resolved completely within four days of conservative treatment. A CT scan of the abdomen demonstrated dilated small-bowel loops measuring up to 3.7 cm, findings consistent with small-bowel obstruction. This complication occurred despite the absence of other medications known to impair gastrointestinal motility, strengthening the temporal association with semaglutide [14]. Another rare case of small-bowel obstruction due to intussusception (where one segment of the intestine telescopes into an adjacent section) was described by Thakkar et al. [14]. The patient, a 32-year-old woman with a past history of iron deficiency anemia, had been receiving subcutaneous 1 mg Ozempic (semaglutide) for 10 months. In the absence of other plausible causes, such as a mass or fecalith, the patient developed intussusception, which subsequently resolved after discontinuing semaglutide. Although our patient received tirzepatide, it belongs to a similar drug class as semaglutide.

Gordon et al. reported a rare but life-threatening case of tirzepatide-associated large-bowel obstruction. Following a recent dose escalation, the 27-year-old patient developed severe gastrointestinal symptoms, and imaging confirmed a high-grade obstruction due to fecalith impaction. Despite conservative measures, she clinically deteriorated and required emergent total colectomy, with pathology demonstrating extensive gangrenous necrosis [15]. In contrast to our patient, this patient had no prior abdominal or pelvic surgeries and no history of inflammatory bowel disease, which could have indicated other potential risk factors for bowel obstruction. This case underscores the potential for tirzepatide to cause severe gastrointestinal dysmotility and fecal stasis, highlighting the importance of vigilance for bowel complications in patients on GLP-1 RAs who present with acute abdominal symptoms [15]. Bowel obstruction in patients receiving GLP-1 RAs has been reported in several studies [10,16,17], without other contributing antimotility agents. Javed et al. described a woman with Roux-en-Y gastric bypass who developed semaglutide-related intussusception, while our patient with adhesive disease on tirzepatide developed a closed-loop obstruction. Though the causes differed, both show how GLP-1 therapies can unmask hidden gastrointestinal risks with serious consequences [18].

These cases further support the possibility that GLP-1 RAs may exacerbate obstruction risk in anatomically predisposed or physiologically vulnerable patients. Although uncommon, these cases highlight a recurrent safety signal suggestive of clinically relevant gastrointestinal motility impairment. Taken together, such evidence strengthens the argument that tirzepatide should carry an explicit warning for bowel obstruction as a potential adverse effect, particularly given its increasing use in diabetes and obesity management.

## Conclusions

This case highlights the complex interplay of multiple risk factors - including prior abdominal surgery, adhesive disease, cirrhosis with ascites, chronic kidney disease, chronic constipation, polypharmacy with constipating agents, and long-term tirzepatide therapy - in the development of severe small-bowel obstruction. The temporal association between tirzepatide initiation, progressive constipation, and subsequent obstruction suggests a possible contributory role of GLP-1 RA-induced motility changes, rather than establishing direct causality. The event was classified as a “possible” adverse drug reaction using the WHO-UMC and Naranjo criteria, reflecting the multifactorial nature of the presentation.

As the use of GLP-1 RAs expands for diabetes and weight management, clinicians should remain vigilant for gastrointestinal symptoms, particularly in patients with overlapping risk factors. Practical risk-mitigation strategies - including baseline assessment of bowel habits, patient education on early warning signs, close monitoring during therapy, and a low threshold for imaging in high-risk individuals - are essential to minimize the risk of severe complications. Ongoing pharmacovigilance and further research are needed to better define the risk profile and mechanisms underlying these rare but serious adverse events, ensuring that the benefits of GLP-1 RA therapy are balanced with patient safety.
